# Evaluation of inpatient services of tertiary comprehensive hospitals based on DRG payment

**DOI:** 10.3389/fpubh.2024.1300765

**Published:** 2024-01-24

**Authors:** Qun-jun Yu, Ya-lin Li, Qin Yin, Ye Lu, Lu-yan Li, Dan-ni Xu, Mei He, Sha Ma, Wu Yan

**Affiliations:** ^1^School of Humanities and Management, Kunming Medical University, Kunming, Yunnan, China; ^2^Department of Pharmacy, Second Affiliated Hospital of Kunming Medical University, Kunming, Yunnan, China; ^3^Clinical Research Center, Children’s Hospital of Nanjing Medical University, Nanjing, Jiangsu, China

**Keywords:** DRG, tertiary comprehensive hospital, inpatient medical service, evaluation, rank sum ratio

## Abstract

**Objective:**

This study aims to evaluate inpatient services in 49 tertiary comprehensive hospitals using indicators from the diagnosis related groups (DRG) payment system.

**Method:**

DRG data from 49 tertiary comprehensive hospitals were obtained from the quality monitoring platform for provincial hospitals, and relevant indicators were identified. The analytic hierarchy process (AHP) was used to compute the weight of each indicator. The rank sum ratio method was used to calculate the weight rank sum ratio (WRSR) value and the corresponding probit value of each hospital. The hospitals were divided into four grades based on the threshold value: excellent, good, fair, and poor.

**Results:**

Eight indicators of the 49 hospitals were scored, and the hospital rankings of indicators varied. The No. 1 hospital ranked first in the indicators of “total number of DRG”, “number of groups”, and “proportion of relative weights (RW) ≥ 2”. The WRSR value of the No.1 hospital was the largest (0.574), and the WRSR value of the No. 44 hospital was the smallest (0.139). The linear regression equation was established: WRSR_predicted_ =-0.141+0.088*Probit, and the regression model was well-fitted (F = 2066.672, *p* < 0.001). The cut-off values of the three WRSRs_predicted_ by the four levels were 0.167, 0.299, and 0.431, respectively. The 49 hospitals were divided into four groups: excellent (4), good (21), average (21), and poor (3). There were significant differences in the average WRSR values of four categories of hospitals (*p* < 0.05).

**Conclusion:**

There were notable variances in the levels of inpatient services among 49 tertiary comprehensive hospitals, and hospitals of the same category also showed different service levels. The evaluation results contribute to the health administrative department and the hospital to optimize the allocation of resources, improve the DRG payment system, and enhance the quality and efficiency of inpatient services.

## 1 Introduction

The issue of healthcare reform and cost control has become a growing concern worldwide. One crucial element that cannot be overlooked is Diagnosis Related Groups (DRG). The DRG payment system, which was first implemented in the United States during the 1980s (1), has gained widespread international adoption for hospital funding (2, 3). This system categorizes patients with similar diagnoses and treatment procedures into specific groups (4), enabling a standardized method for determining payment amounts based on the anticipated resources needed for each case. Numerous countries across the globe have embraced and adapted the DRG payment system, leveraging its proven effectiveness. For instance, Germany implemented DRG in 2003, making it obligatory in 2004. A fundamental aspect of all DRG-based hospital payment systems revolves around the conversion of relative weights into actual payments. This process has contributed to enhancing transparency and fairness in hospital payments to some extent (5, 6). Through extensive analysis of the longitudinal database, Carine Milcent has identified a noteworthy phenomenon: the adoption of more refined DRG classification tended to incentivize upcoding behaviors within hospitals, based on data manipulation, which resulted in an inefficient budget allocation among hospitals (7). Additionally upcoding was another possible mechanism to increase net income (8–10) and is regarded as healthcare fraud (11). In Asia, Peter Leslie Annear et al. examined DRG-based hospital payment systems in Japan, South Korea, and Thailand, pointing out that before DRG be introduced, a country need adequate infrastructure, human resources capacity, information management system, a high degree of hospital autonomy, and sustained levels of government spending, as a result none of these countries introduced a complete DRG system at once, but rather implemented DRG in phases (2). Inke Mathauer and Friedrich Wittenbecher conducted a comprehensive analysis, the DRG implementation in 13 low-and middle-income countries was examined. The study emphasized the significance of technical conditions, including functional IT infrastructure, coding, and costing systems, in determining the suitability of adapting existing DRG models or developing customized approaches. These technical systems played a vital role in facilitating the alignment of the DRG system with the specific circumstances and requirements of each country. Furthermore, the introduction of DRG was also influenced by political dynamics, as stakeholders engaged in lobbying and negotiation processes to safeguard their individual interests (12).

Reform has been extensively implemented in China’s medical and health field, with the reform of medical insurance payment playing a pivotal role (13). Various payment methods, such as fee-for-service, capitation, case-based payment, global budgeting, and DRG, have been explored in many regions in China (14). DRG, recognized internationally as a tool to maintain clinical and resource homogeneity in case mix, has gained significant attention (15, 16). China implemented DRG piloting at the national level in 2017 (17). Currently, the research direction of DRG in China is diverse. For instance, Feng et al. concluded that DRG-based inpatient service management (ISM) played an important role in improving the capacity and efficiency of regional inpatient service in Jiading district in Shanghai (18). Consequently, it was crucial to implement strategies for overseeing and mitigating the adverse ethical consequences and unintended outcomes associated with a case-mix payment system based on DRG in order to guarantee the long-term societal advantages of payment reform within Chinese public hospitals (19).

Although the researches and practice on DRG have been extensive and in-depth in domestical and international level, there are still gaps. This study focuses on evaluating the quality of inpatient services in tertiary comprehensive hospitals in a province of China, which has important practical implications for hospital administrators and policy makers. By understanding hospital inpatient service performance and quality in the context of DRG payments, managers could timely adjust strategies and measures to improve hospital operational efficiency. More importantly, our study helps to enhance the performance and quality of inpatient services in tertiary comprehensive hospitals for the benefits of the general patient population.

## 2 Materials and methods

### 2.1 Research design

The study had a cross-sectional design with data for January 2022. The study was conducted from May 2023 to September 2023.

### 2.2 Data source

In this study, two researchers simultaneously entered data from the Hospital Quality Monitoring and Performance Evaluation Platform (HQMEP) into Excel for consistency comparison and logical check. A multi-indicator comprehensive evaluation of capacity, efficiency, and quality of inpatient service of 49 tertiary comprehensive hospitals in a province was performed as well.

### 2.3 Determination of indicators

The inpatient services of 49 tertiary comprehensive hospitals were evaluated from three aspects: inpatient service capacity, inpatient service efficiency, and inpatient service quality. Inpatient service capacity consists 5 indicators including the total number of DRG, Case Mix Index (CMI), number of groups, proportion of Relative Weights (RW) ≥ 2, and proportion of third- and fourth-level surgeries; inpatient service efficiency consists 2 indicators time consumption index and cost consumption index; and inpatient service quality includes low-risk mortality rate. It is worth noting that the total amount of DRG and the number of DRG groups are important indicators to evaluate the level of hospital’s diagnosis and treatment service, which are evaluated from the perspectives of resource consumption and the scope of diagnosis and treatment services. The total amount of DRG reflects the total amount of hospital services, and the number of DRG groups reflects the breadth and scope of disease coverage of patients admitted to the hospital.

### 2.4 Statistical analysis

To comprehensively evaluate and rank these indicators, we employed the Analytic Hierarchy Process (AHP) and Rank Sum Ratio (RSR) methods. The AHP is a decision-making method proposed by Professor Satty, an American operations researcher at the University of Pittsburgh, in the early 1970s. The AHP combines network system theory and multi-objective comprehensive evaluation methods to provide a hierarchical weighting approach for decision analysis (20). AHP decomposes a problem into different constituent factors based on the nature of the problem and the overall objective to be achieved. These factors are then aggregated and combined into a multi-level analytical structure model, taking into account the interrelationships and hierarchical dependencies among them (21). This ultimately leads to the determination of the relative importance weights or the ranking of alternatives at the lowest level (solution level) relative to the highest level (objective level). In this study, the AHP method was employed to determine the relative weight of each indicator and establish a comprehensive evaluation model for hospital inpatient services. The construction of a performance evaluation indicator system for inpatient medical services of tertiary comprehensive hospitals based on the AHP is presented in Table 1.

**Table 1:** A performance evaluation indicator system for inpatient medical services of tertiary comprehensive hospitals.

Target level (A)	Normative level (B)	Program level (C)			
		Indicator	Proportion of weight	Weights	Property of indicator
Inpatient service performance	Inpatient service capacity (0.47)	Total number of DRG	0.2	0.094	High-performance indicator
		CMI	0.5	0.235	High-performance indicator
		Number of groups	0.1	0.047	High-performance indicator
		Proportion of RW ≥ 2	0.1	0.047	High-performance indicator
		Proportion of third- and fourth-level surgeries	0.1	0.047	High-performance indicator
	Inpatient service efficiency (0.24)	Time consumption index	0.5	0.120	Low-performance indicator
		Cost consumption index	0.5	0.120	Low-performance indicator
	Inpatient service quality (0.29)	Low-risk mortality rate	1	0.290	Low-performance indicator

A single ranking and consistency test were conducted for the A to B level, and the maximum eigenvalue was found to be 3.007. Referring to the RI table, the corresponding RI value was 0.525. Therefore, the consistency ratio (CR) was calculated as CI/RI = 0.006, which is less than 0.1, indicating that the test passed the consistency check. Additionally, a total ranking and consistency test were performed for the B to C level using the same method, which the result showed a CR value of 0.001, and is also less than 0.1, indicating that the test also passed the consistency check.

The Rank Sum Ratio (RSR) method was proposed by Professor Fengtiao Tian from China (22). This method is a statistical analysis approach that combines the strengths of classical parametric statistics and modern nonparametric statistics. It is extensively applied in fields like healthcare, technology, and economics (23). One of the key advantages of the RSR method is its reliance on nonparametric techniques, which allows for evaluating a wide range of objects without the need for specific indicator selection (24). The RSR method follows a general process. Firstly, the High-performance indicators are sorted from small to large, while the low-performance indicators are sorted from large to small. Then, the rank sum ratio is calculated to obtain the dimensionless statistic WRSR. Afterward, the distribution of WRSR is determined using the concepts and methods of parametric statistical analysis. Finally, the obtained WRSR value from the regression equation is used to directly rank or classify the evaluation objects, enabling a comprehensive evaluation of these objects. This study referred to the four-level Probit threshold (3.5, 5, 6.5) recommended by Professor Tian (22).

It should be noted that in this study, a non-integer rank method was employed for ranking. This method was similar to linear interpolation and had the advantage of accurately reflecting the relative differences in size between the original data. It also minimized the loss of information regarding the relative differences in the size of the original data. In addition, the non-integer rank method established a quantitative linear correspondence between the ranked values and the original data, making it superior to the integer rank method.

Besides, to further validate the rationality of the classification results, it was necessary to conduct a test to verify if there was a significant difference in the overall mean of the WRSR values of hospitals in each category, including examining whether the WRSR values of each hospital in the four categories met the conditions of variance homogeneity and whether they followed a normal distribution. If both conditions were met, a one-way analysis of variance (ANOVA) would be used to test the rationality of the classification results. Otherwise, a non-parametric test for multiple independent samples would be employed. A *p* value <0.05 based on 2-tailed test results should be considered statistically significant. All analyses were performed with Excel 2019 and SPSS 26.0 software.

## 3 Results

### 3.1 Ranking results of the evaluation indicator system for inpatient medical service performance based on non-integer ranks

The original data of eight indicators from 49 tertiary comprehensive hospitals were input into the non-integer rank-based formula, which generated rank scores for each indicator of all hospitals. These rank scores are presented in Table 2.

**Table 2:** The rank scores of the performance evaluation indicator system for inpatient medical services of tertiary comprehensive hospitals.

ID	Total number of DRG	CMI	Number of groups	Proportion of Relative Weights (RW) ≥ 2	Proportion of third- and fourth-level surgeries	Low-risk mortality rate	Time consumption index	Cost consumption index
1	49.00	48.18	49.00	49.00	44.79	1.00	36.03	7.40
2	35.19	31.43	43.03	30.90	44.16	1.00	33.43	4.84
3	30.65	23.57	42.04	23.81	31.56	1.00	24.35	19.56
4	20.56	6.33	27.98	6.37	8.58	1.00	41.22	43.88
5	31.14	49.00	40.05	38.46	46.89	1.00	36.03	3.56
6	22.84	19.80	34.20	20.14	25.68	1.00	11.38	9.32
7	20.41	15.58	26.62	13.57	7.31	1.00	32.14	41.32
8	19.29	12.87	34.20	14.75	28.45	1.00	12.68	18.28
9	21.56	30.07	39.80	26.66	28.53	1.00	15.27	21.48
10	20.16	30.91	34.33	30.60	30.21	1.00	33.43	18.92
11	18.53	22.84	36.56	22.66	25.85	1.00	15.27	22.12
12	18.56	24.07	30.84	18.69	33.08	1.00	20.46	11.24
13	19.54	29.66	35.69	29.81	38.34	1.00	19.16	16.36
14	21.34	40.46	29.48	38.84	49.00	1.00	30.84	8.04
15	17.10	20.80	34.82	20.47	26.62	1.00	23.05	17.00
16	13.52	10.75	26.12	9.52	22.90	1.00	23.05	29.80
17	16.03	27.12	31.22	24.22	29.72	1.00	21.76	13.80
18	11.25	1.00	21.64	2.04	6.46	1.00	49.00	49.00
19	13.18	14.39	25.50	13.38	7.82	1.00	12.68	43.88
20	12.91	15.71	28.36	18.06	18.66	1.00	21.76	17.64
21	16.11	41.63	27.36	32.35	41.76	1.00	24.35	1.00
22	14.71	32.16	32.96	30.85	37.48	1.00	34.73	15.08
23	10.06	1.95	16.17	1.00	14.08	1.00	21.76	41.96
24	11.11	13.38	23.51	14.94	15.18	1.00	38.62	34.92
25	9.58	8.99	14.43	5.90	1.00	1.00	23.05	43.88
26	9.15	9.27	22.51	7.30	9.53	1.00	25.65	38.76
27	10.32	19.01	24.25	15.13	34.27	1.00	10.08	11.24
28	8.72	7.37	17.54	4.09	17.92	1.00	12.68	20.20
29	9.91	18.02	26.74	17.54	31.05	1.00	8.78	8.68
30	10.33	23.17	22.26	19.81	7.71	1.00	25.65	40.04
31	9.39	19.00	23.76	20.00	32.36	1.00	19.16	18.28
32	7.90	7.07	10.45	1.47	6.80	1.00	28.24	42.60
33	7.83	11.70	18.04	6.72	26.15	1.00	36.03	33.64
34	8.19	15.43	19.65	14.03	20.31	1.00	33.43	34.92
35	9.42	30.57	21.15	36.13	34.39	1.00	41.22	33.64
36	8.32	21.03	24.01	24.69	40.72	1.00	16.57	18.28
37	7.05	10.53	18.53	7.76	9.29	1.00	17.86	33.00
38	7.82	19.07	14.18	11.46	14.79	1.00	24.35	32.36
39	8.70	34.88	21.02	26.16	39.09	1.00	19.16	6.76
40	6.19	10.25	15.67	12.94	5.66	1.00	42.51	39.40
41	5.50	12.19	17.79	11.41	12.57	1.00	34.73	29.80
42	4.67	7.58	8.09	1.60	10.39	1.00	16.57	40.68
43	4.15	4.43	12.69	5.96	11.81	1.00	37.32	31.08
44	4.23	10.09	7.84	15.92	17.80	1.00	1.00	13.80
45	3.58	3.88	12.94	1.82	10.28	1.00	19.16	37.48
46	3.58	5.15	12.07	5.24	14.65	1.00	7.49	32.36
47	3.14	14.46	9.95	14.83	5.59	1.00	32.14	33.00
48	2.52	5.18	5.48	6.12	12.39	1.00	16.57	34.92
49	1.00	10.02	1.00	8.17	14.54	1.00	30.84	22.12

To indicate low-risk mortality, all ranks of this indicator were coded as 1.00, as each hospital had a 0 value for it.

The ranking of hospitals varied across different indicators. In terms of the “Total number of DRG” indicator, Hospital No. 1 ranked first, while Hospital No. 49 ranked last. For the “CMI” indicator, Hospital No. 5 ranked first, while Hospital No. 18 ranked last. Hospital No. 1 also ranked first in the “Number of groups,” while Hospital No. 49 ranked last. Hospital No. 1 ranked first in the “Proportion of Relative Weights (RW) ≥ 2,” while Hospital No. 23 ranked last. On the other hand, Hospital No. 14 ranked first in the “Proportion of third- and fourth-level surgeries” indicator, while Hospital No. 25 ranked last. When it came to the “Time consumption index,” Hospital No.44 ranked first, while Hospital No. 18 ranked last. Hospital No. 21 ranked first in the “Cost consumption index” indicator, while Hospital No. 18 ranked last. Notably, Hospital No. 1 ranked first in three indicators (“Total number of DRG,” “Number of groups,” and “Proportion of Relative Weights (RW) ≥ 2”), while Hospital No. 18 ranked last in three indicators (“CMI,” “Time consumption index” and “Cost consumption index”).

### 3.2 WRSR values and their distribution

The WRSR value of each hospital can be calculated by substituting the weight of each indicator and the rank of the corresponding indicator of each hospital into the formula of weighted rank-sum ratio. Then, we obtained the downward cumulative frequency of ∑f/n × 100 (expressed in %) and its corresponding probit value.

**Table 3:** Distribution of WRSR for inpatient medical services of tertiary comprehensive hospitals.

ID	WRSR	*f*	*∑f*	*R*	$\bar{R}$	∑f/n × 100	Probit	WRSR _predicted_
44	0.138528	1	1	1	1	2.040816	2.954609	0.11893
46	0.165696	1	2	2	2	4.081633	3.258709	0.14568
28	0.176448	1	3	3	3	6.122449	3.455425	0.16298
48	0.184683	1	4	4	4	8.163265	3.605827	0.17621
45	0.194141	1	5	5	5	10.20408	3.729992	0.18712
49	0.208316	1	6	6	6	12.2449	3.837169	0.19655
42	0.210673	1	7	7	7	14.28571	3.932429	0.20493
23	0.220575	1	8	8	8	16.32653	4.018874	0.21253
29	0.226373	1	9	9	9	18.36735	4.098546	0.21954
37	0.228637	1	10	10	10	20.40816	4.17287	0.22607
43	0.231854	1	11	11	11	22.44898	4.242883	0.23223
27	0.239739	1	12	12	12	24.4898	4.309367	0.23808
32	0.24641	1	13	13	13	26.53061	4.372928	0.24367
25	0.251768	1	14	14	14	28.57143	4.434051	0.24904
8	0.254688	1	15	15	15	30.61224	4.493128	0.25424
26	0.263393	1	16	16	16	32.65306	4.550486	0.25928
20	0.264931	1	17	17	17	34.69388	4.606402	0.2642
16	0.269005	1	18	18	18	36.73469	4.661112	0.26901
47	0.26992	1	19	19	19	38.77551	4.714825	0.27374
6	0.272128	1	20	20	20	40.81633	4.767728	0.27839
41	0.273024	1	21	21	21	42.85714	4.819988	0.28298
31	0.27976	1	22	22	22	44.89796	4.87176	0.28754
19	0.28352	1	23	23	23	46.93878	4.923191	0.29206
38	0.290066	1	24	24	24	48.97959	4.974419	0.29657
36	0.293822	1	25	25	25	51.02041	5.025581	0.30107
33	0.296488	1	26	26	26	53.06122	5.076809	0.30557
40	0.300406	1	27	27	27	55.10204	5.12824	0.31009
18	0.301211	1	28	28	28	57.14286	5.180012	0.31465
12	0.313828	1	29	29	29	59.18367	5.232272	0.31924
34	0.314824	1	30	30	30	61.22449	5.285175	0.3239
15	0.315164	1	31	31	31	63.26531	5.338888	0.32862
24	0.322935	1	32	32	32	65.30612	5.393598	0.33343
11	0.32418	1	33	33	33	67.34694	5.449514	0.33835
4	0.325312	1	34	34	34	69.38776	5.506872	0.34339
17	0.335488	1	35	35	35	71.42857	5.565949	0.34859
39	0.33613	1	36	36	36	73.46939	5.627072	0.35396
7	0.345229	1	37	37	37	75.5102	5.690633	0.35955
30	0.345473	1	38	38	38	77.55102	5.757117	0.3654
13	0.372255	1	39	39	39	79.59184	5.82713	0.37156
9	0.37259	1	40	40	40	81.63265	5.901454	0.37809
3	0.378701	1	41	41	41	83.67347	5.981126	0.3851
21	0.39591	1	42	42	42	85.71429	6.067571	0.3927
22	0.40752	1	43	43	43	87.7551	6.162831	0.40108
10	0.412297	1	44	44	44	89.79592	6.270008	0.41051
2	0.431156	1	45	45	45	91.83673	6.394173	0.42143
35	0.441861	1	46	46	46	93.87755	6.544575	0.43465
14	0.448642	1	47	47	47	95.91837	6.741291	0.45195
5	0.517886	1	48	48	48	97.95918	7.045391	0.4787
1	0.574321	1	49	49	49	99.4898	7.568835	0.52473

The *f* represents the frequency of occurrence of the corresponding value of WRSR, ∑*f* is the cumulative frequency of each WRSR value, *R* is the rank of the corresponding WRSR value, $\bar{R}$ is the average rank corresponding to each WRSR value, ∑f/n × 100 represents the cumulative frequency in percentage and the Probit is the value corresponding to the cumulative frequency.

Based on the calculation results in Table 3, a linear regression equation was formulated with Probit as the independent variable and WRSR as the dependent variable.

The results indicated that the model had a strong correlation with *R* = 0.989 (*p* < 0.001). The *t*-statistic for the independent variable Probit was 45.461 and *p* < 0.05, suggesting a significant linear relationship between Probit and WRSR. The regression equation was WRSR_predicted_ = −0.141 + 0.088 Probit (*F* = 2066.672, *p* < 0.001). Furthermore, since the regression model was well-fitted, subsequent categorization could be conducted.

### 3.3 Categorization result of general evaluation of inpatient medical services in tertiary comprehensive hospitals

The result ranked 49 hospitals and divided them into 4 grades: excellent, good, average, and poor. Referring to the four level thresholds, the corresponding results were as follows:

According to probit = 3.5, the WRSR_predicted_ was calculated as -0.141 + 0.088 × 3.5 = 0.167.
According to probit = 5, the WRSR_predicted_ was calculated as 0.299.
According to Probit = 6.5, the WRSR_predicted_ was calculated as 0.431.

After conducting the homogeneity of variance test for WRSR values among hospitals in the four categories, the *p*-value was 0.062, which suggesting that the variance homogeneity condition was satisfied. Furthermore, the normal distribution test for WRSR values showed the significance levels for all hospitals in each category indicating that the WRSR values followed a normal distribution in each category. Additionally, pairwise comparisons revealed that the mean WRSR values of the four categories differed significantly. These findings demonstrated that the classification results presented in Table 4 were statistically significant and reasonable.

**Table 4:** Ranking and categorization of the comprehensive evaluation of inpatient medical services of tertiary comprehensive hospitals.

Categorization	P	Probit	WRSR_predicted_	ID
excellent	≥P93.319	≥6.5	≥0.431	1, 5, 14, 35
good	P50 ~ <P93.319	5 ~ 6.5	0.299 ~ 0.431	2, 10, 22, 21, 3, 9, 13, 30, 7, 39, 17, 4, 11, 24, 15, 34, 12, 18, 40, 33, 36
average	P6.681 ~ <P50	3.5 ~ 5	0.167 ~ 0.299	38, 19, 31, 41, 6, 47, 16, 20, 26, 8, 25, 32, 27, 43, 37, 29, 23, 42, 49, 45, 48
poor	<P6.681	<3.5	<0.167	28, 46, 44

## 4 Discussion

The implementation of DRG payment reform has necessitated hospitals to adopt more sophisticated management practices. This reform has posed significant challenges and impacts on hospital management, particularly in terms of restricting the growth of hospital medical income due to the pre-payment system enforced by DRG. As a result, it has become crucial to control disease costs. To assist hospitals in refining their management, a quantitative evaluation and assessment of inpatient service levels in tertiary comprehensive hospitals in a specific province in China could be an effective approach. By determining the weight of relevant indicators and utilizing the RSR method, the WRSR values of 49 tertiary comprehensive hospitals were calculated to determine the hospital ranking results. These results were then classified into 4 categories, ultimately providing an overall ranking of tertiary comprehensive hospitals in the province.

Hospitals in the “good,” “average,” and “poor” categories should carefully study the successful experiences and practices of four “excellent” hospitals to improve their inpatient services. Similarly, these four hospitals would learn from and adopt the experiences and practices of higher-ranked hospitals in the country to further solidify their leading position within the province. Moreover, our study may promote hospital administrators understand their ranking status and enhance willingness to learn from higher-ranked hospitals, to boost their motivation and sense of responsibility and enhance their awareness of quality and efficiency when providing medical services, ultimately improving the overall level of inpatient care. Furthermore, the sorting and categorization results can also be used as a reference by health administrative departments to evaluate and assess hospitals. This information can aid in the optimal allocation of health resources and government spending, thereby improving the development of the provincial health industry and providing higher-quality health services to the population.

The quality of the data on the home page of medical records and the accuracy of the coding has significant impacts on the accuracy of DRG grouping. The validity and accuracy of the sorting and classifying of 49 tertiary comprehensive hospitals in a Chinese province rely heavily on the accuracy of DRG grouping data. The quality of DRG grouping data is highly dependent on the level of data on home page of medical records and accuracy of diagnostic coding. Therefore, accurate completion of the home page of medical records and precise diagnostic coding has crucial for ensuring the quality assessment of medical services and facilitating payment and settlement of medical insurance.

Some studies have expressed similar views. For instance, Peter Leslie Annear et al. conducted research on the DRG systems in Japan, Korea, and Thailand and emphasized that “the accuracy of coding, including the adequacy of documentation in clinical records, is essential” (2). Additionally, based on DRG implemented in a private healthcare institution, Venancio García Calderón et al. believed through more efficient and accurate coding, DRG was useful within the institution to generate indicators on resources, cost, length of stay, and goals for each service (25). It emphasized the quality and coding accuracy of medical record homepage, because it involves the cooperation between medical personnel, coding personnel, and medical insurance staff. Their business familiarity and cooperation will affect the accuracy of coding and DRG grouping. By accurately recording and classifying patient conditions, hospitals administrators can assess their medical services, gain insight into patient conditions and treatment outcomes, and promptly identify and address existing issues. This process ultimately enhances the overall quality of medical care. Many hospital manager now recognize the importance of quality control and accuracy in the diagnostic coding of the home page of medical records in the DRG payment system. To achieve this, they have implemented various effective approaches. Numerous medical staff, coders, and IT personnel receive regular or occasional training to ensure they are up to date with the latest specifications for completing and coding the home page of medical records. Furthermore, efforts have been made to enhance the coders’ medical knowledge. Some hospitals have even established specific regulations to supervise medical staff and ensure that the home page of medical records is filled out completely, accurately, and legibly.

The utilization of AI technology to enhance the accuracy of diagnostic coding is expected to foster a consensus among hospital management and motivate them to prioritize correct coding practices (26–28), which will aid medical and coding staff in conducting more detailed analyses and categorizations of diseases. It will eventually decrease their workload, enhance efficiency, and minimize error rates.

The significance of integrating business and finance is becoming more evident as the ranking and classification of the 49 tertiary comprehensive hospitals consider not only the quality and efficiency of their inpatient services, but also their financial operations. Therefore, the integration of business and finance in hospitals plays a crucial role in this context (29). The integration of business and finance aims to achieve an intensive integration of the business and financial activities within hospitals. This integration encompasses processes, data, and management, and highlights the significant role of financial management in the overall business operations. Similarly, it emphasizes the necessity of business activities in effectively managing finance. Within the framework of a modern hospital governance system, the integration of business and finance holds significant value in enhancing operational efficiency and improving the quality of services provided by hospitals. The four hospitals in the “excellent” category are at the forefront in terms of Total number of DRG, CMI, cost consumption index, and so on. It is highly probable that they have streamlined their healthcare processes through “business-finance integration” and conducted profound research on the cost consumption index, which enables them to effectively manage their cost consumption index. Moreover, these hospitals exhibit a positive and mutually beneficial relationship between financial performance and the DRG index. Hospitals graded as “average” or “poor” may encounter challenges in achieving “integration of business and finance.” These challenges include the presence of an information island caused by departmental barriers (30), and the absence of multi-disciplinary personnel for hospital operations managers (31). These factors might have an impact on the evaluation ranking of inpatient services in these hospitals to some extent. In short, in the process of integrating business and finance, hospitals are expected to achieve mutual promotion of business and finance, thereby improving hospital services, which is beneficial for hospitals to achieve the goal of modern management.

The differences in the qualities of medical services among the 49 tertiary hospitals are not only caused by the hospitals’ resources, but also closely related to the socio-economic factors of their respective regions, such as economic level and population. In particular, the hardware and software resources of hospitals in regions with higher economic development levels are often superior. In addition, the total population is also a crucial factor affecting the development of tertiary hospitals. Regions with larger populations provide hospitals with a greater number of disease cases, facilitating the expansion of these regional hospitals may help to improve their medical standards. Furthermore, the adjustment of health policies is also an important factor. For instance, the adjustment of medical and health policies will lead to changes in the hospital’s financial revenue and expenditure; The adjustment of medical service prices by the government pricing authorities or the reform of medical insurance payment methods may also have a profound impact on the revenue source and cost structure of hospitals, and then affect the operation and service level of hospital.

This paper presented a study that ranked and classified 49 tertiary comprehensive hospitals in a province of China based on DRG indicators using data from the information platform. The results of the study provided valuable insights for the improvement of hospitals and the development of health policies in the province. However, there were some limitations in the study. Firstly, this study used monthly data, which may be affected by seasonal factors or other variables, and in turn cannot accurately capture long-term trends in inpatient services across hospitals. Future research could explore the use of longer time span data such as quarterly or annual data to more accurately assess the comprehensive performance of hospital inpatient services. In addition, we included 8 indicators of inpatient service performance, there were still important indicators that have not been included due to the accessibility of data. DRG enrollment rate, patient satisfaction and patient complaint rate are also important indicators in evaluating the quality of hospital inpatient services. The inclusion of these indicators would enrich and optimize the evaluation system in multiple dimensions, resulting in more comprehensive and objective evaluation results. Additionally, it would provide hospital management and health administrative authorities with better decision-making information.

## 5 Conclusion

The study revealed notable variations in the provision of inpatient services among tertiary comprehensive hospitals in the DRG payment system. Significant differences were observed in indicators between hospitals of different categories, and perhaps there may be some differences in the level of inpatient services among hospitals within the same category. The findings could provide hospital managers with a comprehensive and fair understanding of the industry landscape and the position of their own hospitals. It highlights the importance of core indicators, which could be used to adjust hospital resource allocation, improve management practices, regulate medical service behavior, optimize medical processes, and enhance the overall quality of medical care.

## Data availability statement

The raw data supporting the conclusions of this article will be made available by the authors, without undue reservation.

## Author contributions

Q-jY: Conceptualization, Methodology, Writing – review & editing, Writing – original draft. Y-lL: Conceptualization, Methodology, Writing – review & editing, Formal analysis. QY: Conceptualization, Data curation, Resources, Software, Writing – review & editing. YL: Conceptualization, Data curation, Investigation, Software, Writing – review & editing. L-yL: Conceptualization, Data curation, Funding acquisition, Writing – review & editing. D-nX: Conceptualization, Funding acquisition, Investigation, Writing – review & editing. MH: Conceptualization, Funding acquisition, Investigation, Methodology, Writing – review & editing. SM: Conceptualization, Data curation, Investigation, Writing – original draft. WY: Conceptualization, Formal analysis, Software, Supervision, Writing – original draft, Writing – review & editing.
